# Disability and Anxiety in Vestibular Diseases: A Cross-Sectional Study

**DOI:** 10.7759/cureus.11813

**Published:** 2020-11-30

**Authors:** Arash Bayat, Reza Hoseinabadi, Nader Saki, Roya Sanayi

**Affiliations:** 1 Hearing Research Center, Ahvaz Jundishapur University of Medical Sciences, Ahvaz, IRN; 2 Department of Audiology, School of Rehabilitation, Tehran University of Medical Sciences, Tehran, IRN

**Keywords:** anxiety, vestibular diseases, dizziness, vertigo, benign paroxysmal positional vertigo, meniere’s disease, unilateral weakness, central vertigo

## Abstract

Introduction

Patients with dizziness and vertigo usually experience psychological, physical, and social functioning limitations that may affect their daily living activities. In order to better understand disability and anxiety in patients with vertigo, in the present study we aimed to investigate the correlation between disability and anxiety in four different types of diseases causing vertigo. Moreover, the difference between the observed disabilities in these etiologies of vertigo was studied.

Materials and methods

In this analytic cross-sectional design, 130 patients (52 male, 78 female; age range: 18-75 years) with dizziness/vertigo who were referred to our balance clinic participated. All patients underwent a detailed diagnostic procedure including neurological, clinical, and otological evaluations. Dizziness Handicap Inventory (DHI) and the Beck Anxiety Inventory (BAI) were used to assess handicap and anxiety, respectively.

Results

There were no significant differences in “total DHI” and DHI subcomponent scores among different study populations (p>0.05). In terms of the BAI score, the one-way analysis of variance (ANOVA) test indicated no significant differences among the four groups (p=0.158). Our results exhibited a significant positive correlation between the BAI and “total DHI” and “DHI subcomponents” values.

Conclusion

The degree of disability and anxiety is not different between patients with Benign paroxysmal positional vertigo (BPPV), Meniere’s disease (MD), unilateral weakness (UW), and central causes. The significant positive correlation between the BAI and “total DHI” and “DHI subcomponents” values shows that the possibility of anxiety in patients with vertigo should not be ignored.

## Introduction

Dizziness is a common clinical symptom, with a lifetime prevalence of about 25-30% in the adult population. Dizziness is usually characterized by an individual's perceived sense of spinning motion, loss of balance, or feelings of lightheadedness [1,2]. It has been documented that various otologic and non-otologic disorders could lead to vertigo or dizziness sensation including Benign paroxysmal positional vertigo (BPPV), vestibular neuritis (VN), Meniere’s disease (MD), vestibular migraine (VM), or psychological problems [3,4].

It has been suggested that patients with dizziness and vertigo usually experience physical and social functioning limitations that may affect their daily living activities [5]. Many patients with vertigo and dizziness become physically inactive and withdraw from their social lives [2,6,7]. In numerous studies, disability and handicap have been addressed as major consequences of vestibular diseases [8-10]. It has been shown that it is not possible to identify recovery of patients following acute vestibular disorders using vestibular tests and therefore, other tools are required to achieve this aim [11-13]. Taken together, quality of life (QoL) can be markedly impaired as the result of dizziness [14] especially vestibular dizziness [15]. Moreover, patients with dizziness also are affected by functional disability [16], depression, poor participation in social activities, reduced health level and falling [17]. Early diagnosis and management of patients with vertigo and dizziness symptoms are of great importance to reduce disability and negative effects of dizziness on patients. Therefore, being aware of the negative effects of different vestibular disorders on the QoL of vertiginous patients constitute an important part of vestibular rehabilitation programs.

It has been shown that many patients with vestibular dysfunctions also have depression/anxiety, which suggests a possible somatopsychic contribution to vertigo or dizziness. The coexistence of these problems could lead to a vicious circle and have a noticeable impact on their therapeutic outcome and quality of life [13]. Vestibular disorders may trigger or cause anxiety because of dysfunctional circuitry in some areas including the amygdale, hippocampus, and infralimbic cortex. On the other hand, individuals with psychiatric problems often report subjective dizziness or unsteadiness as a concomitant complication in their illness. Therefore, it has been hypothesized that there is a reciprocal association between the vestibular and emotional processing systems [18]. 

In a study done by Best et al., it was indicated that the amount of vestibular involvement did not associate with the psychological strain of subjects who suffer from vestibular dysfunctions, not with dizziness-induced anxiety, disease-specific handicap, subjective symptom severity, or emotional distress. Instead, the extent of fluctuations of vestibular problems more than one-year follow-up associated positively with the degree of subjectively reported dizziness symptoms [13]. Teggi et al. study demonstrated that following acute vestibular loss, vertiginous patients often complain from anxiety or depression and may manifest a poor improvement, and a high persistency of psychiatric symptoms [19].

The purpose of this study was to evaluate the disability and psychological distress (anxiety) levels in patients with different vestibular disorders. Furthermore, we decided to determine factors modifying the development of dizziness disability in these patients, including age, gender, the degree of anxiety, and type of vestibular disorder.

## Materials and methods

Participants

The participants of the present analytic cross-sectional study were 130 patients (52 male, 78 female; age range: 18-75 years) who visited our “balance clinic” between August 2019 and May 2020 complaining of dizziness/vertigo. Following a detailed case-history, all participants underwent a diagnostic procedure including neurological, clinical, and otological evaluations. The diagnosis of vestibular dysfunctions was based on the positional and positioning examinations, air caloric test, and vestibular evoked myogenic potentials test. Furthermore, a complete blood count/chemistry and brain imaging tests were conducted. Patients with a history of previous psychological disorders or suspicion of psychogenic dizziness were excluded.

Procedures

Patients’ dizziness and psychological symptoms were evaluated using the Persian versions of the Dizziness Handicap Inventory (DHI) [20] and the Beck Anxiety Inventory (BAI) [21], respectively.

The BA) is a 21-item self-administered scale developed to measure emotional, physiological, and cognitive-related symptoms of anxiety. Each item is a clear description of a symptom of anxiety in one of its four major aspects: (1) subjective (e.g., unable to relax), (2) neurophysiologic (e.g., numbness or tingling), (3) autonomic (e.g., feeling hot), or (4) panic-related (e.g., fear of losing control). Each item is scored on a four-point Likert scale: not at all (score=0); mildly (score=1); moderately (score=2), and severely (score=4). BAI total scores range from 0 to 63, a higher score denotes a higher level of anxiety. Total scores of 0-7 indicate “minimal anxiety”, 8-15 “mild anxiety”, 16-25 "moderate anxiety", and 26-63 "severe anxiety". Item responses are examined to determine whether the symptoms appear mostly subjective, neurophysiologic, autonomic, or panic-related [22].

The DHI is a self-reported scale to investigate handicapping consequences imposed by vestibular disorders. This scale describes three aspects of a patient’s daily living, including emotional problems (nine items), functional problems (nine items), and physical problems (seven items). The DHI is a 25-item questionnaire, with a total score between 0 and 100. Higher scores represent a greater level of dizziness in daily life. Scores up to 30 represented a “mild handicap”, scores between 31 and 60 indicate a “moderate handicap”, and scores of more than 60 represented a “severe handicap” [23].

Statistical analysis

Statistical package for the social sciences (SPSS) 22.0 software (SPSS Inc, Chicago, Illinois) was used for data analysis. Data with a normal distribution are presented as mean ± standard deviation (SD). One-way analysis of variance (ANOVA) test was utilized for the analyses of numeric data with a normal distribution (age, BAI score, and DHI score) among different study groups. Differences were deemed to indicate statistical significance at p<0.05.

## Results

The average age of the participants was 48.13 years, and the female/male ratio was 46:23. Of the 130 patients, 41 patients with BPPV, 29 with MD, 38 with UW, 22 with central vertigo were diagnosed. The demographic characteristics of participants are summarized in Table 1. We found no statistical differences between age (ANOVA test; p=0.707) and gender (Chi-square test; p=0.253) of the participants among different study groups.

**Table 1:** The mean DHI scores according to the disease groups (n=130) DHI: Dizziness Handicap Inventory, BPPV: Benign paroxysmal positional vertigo, UW: unilateral weakness

Parameter	Vestibular Disorder						p-Value
	Central (n=22)		UW (n=38)	Meniere’s (n=29)		BPPV (n=41)	
DHI-Physical	13.09 ± 11.85	15.87 ± 9.82		17.66 ± 7.76	17.56 ± 7.76		0.564
DHI-Emotional	8.45 ± 8.11	12.69 ± 8.45		11.01 ± 5.49	12.68 ± 9.04		0.499
DHI-Functional	13.81 ± 11.43	15.31 ± 8.87		16.16 ± 9.55	17.56 ± 8.35		0.343
DHI-Total	35.45 ± 29.98	43.86 ± 24.41		44.83 ±21.15	47.82 ± 21.38		0.707

ANOVA test revealed that the comparison of total DHI score did not produce significant results among different disease groups (p=70.7). Furthermore, the comparison of DHI subscales was not statistically different across different vestibular etiologies (p=0.564 for physical, p=0.564 for emotional, and p=0.343 for functional subscales) (Table 2). 

**Table 2:** Demographic characteristics of patients with vestibular disorders (n=130) BPPV: Benign paroxysmal positional vertigo, UW: unilateral weakness; M: male; F: female

Parameter	Vestibular Disorder				
	Central (n=22)	UW (n=38)	Meniere’s (n=29)		BPPV (n=41)
Age	48.66 ± 22.18	48.38 ± 13.20	44.81 ± 13.28	49.88 ± 15.06	
Gender	10 M; 12 F	14 M; 24 F	11 M; 18 F	17 M; 24 F	

Analysis of variance of the BAI scores showed no significant differences among the four groups of vestibular disorders (p=0.158). However, there was no significant statistical difference in the BAI scores among the different groups (Table 3).

**Table 3:** The mean BAI scores according to the disease groups (n=130) BAI: Beck Anxiety Index, BPPV: Benign paroxysmal positional vertigo, UW: unilateral weakness

Parameter	Vestibular Disorder				p-Value
	Central (n=22)	UW (n=38)	Meniere’s (n=29)	BPPV (n=41)	
BAI	10.82±12.67	19.34±10.58	21.16±10.91	19.17±13.12	0.158

We observed a significant positive correlation between the scores of BAI and DHI scales (Table 4). Furthermore, logistic regression analysis was performed with gender, age, total DHI score, and the types of vestibular disorder as modifying variables for the development of psychological anxiety. Our finding indicated that only the total DHI score was associated significantly with the BAI scores of individuals with the vestibular disorder (p<0.001).

**Table 4:** Correlation between DHI and BAI scores BAI: Beck Anxiety Index; DHI: Dizziness Handicap Inventory; r: Pearson’s correlation coefficient

Parameter	DHI			
	DHI-Physical	DHI-Emotional	DHI-Functional	DHI-Total
BAI	r=0.511; p<0.001	r=0.649; p<0.001	r=0.556; p<0.001	r=0.636; p<0.001

## Discussion

The current study showed that dizziness and vertigo resulting from different vestibular disorders negatively affected the QoL of patients in their daily living activities. The vertiginous symptoms reported by our respondents were at a moderate level in all groups, indicating that all of these disorders have a similar impact on dizzy patients.

In all groups, the least compromised subscale of the DHI was the emotional subscale. It has been shown that the emotional component is related to the psychological aspects of dizziness, such as how subjects feel about the opinions of others or depression issues [23-25]. Our results showed that a longer duration of a vestibular disorder is associated with greater emotional involvement. In our study, we assessed the patients after their diagnosis, and therefore the duration of the disease was rather short. Hence, the impact of the vestibular disorder on the patient’s emotions and feelings was less than the functional and physical aspects.

It has been reported that nearly half of patients referring to specialized dizziness clinics show high comorbidity with some psychiatric disorders, such as depression and anxiety [26]. In our study, patients with central vertigo exhibited a mild degree of anxiety, while patients with other vestibular etiologies showed a moderate level of anxiety. It has been suggested that the most important factor affecting the development of psychological distress in patients with vestibular disorder is the severity of vertigo symptoms, rather than the type of vestibular dysfunction. The experience of dizziness or vertigo symptoms exerts considerable stress on affected patients, which may induce psychological distress regardless of the pathophysiological mechanism of a specific vestibular disease [27].

The comparison of BAI scores in different study groups was not statistically different. It shows that the degree of anxiety across different vestibular diseases was rather similar. Our results confirmed the comorbidity of anxiety and dizziness. It has been shown that psychological distress is significantly elevated in patients with VM, MD, and primary psychiatric dizziness, but it is independent of the amount of vestibular deficit [28]. We did not find any difference between different etiologies. A previous study showed no correlation between the acute or chronic vestibular disorder and pathologies on psychometric testing, but subgroups of patients with Meniere’s disease and vestibular migraine had high anxiety and depression scores [29]. In our study, we did not find any difference between MD and other vestibular disorders in terms of anxiety.

Our results revealed that the total DHI score was associated significantly with the BAI scores of individuals with a vestibular disorder. This shows that the more severe is the disability of a patient with vertigo, the more would be the degree of anxiety. In another study, it has been shown that DHI scores and psychological distress were closely associated [27]. A number of theories have been proposed to explain the relationship between anxiety and the perceived severity of dizziness. It seems that high anxiety and autonomic symptom levels might be representative of physiological arousal level or even hyperventilation. In fact, objective physiological evaluations of respiratory rate in subjects with dizziness have shown that increased somatization scores are associated with changes in respiration rate following head movements which may lead to dizziness. Arousal and hyperventilation factors may directly enhance unsteadiness and disorientation via the various reciprocal neuronal connections between the cerebellum, vestibular system, and autonomic brainstem structure [3,30].

## Conclusions

Our results demonstrated that disability levels and psychological symptoms did not differ significantly among various vestibular abnormalities. A significant correlation between the perceived dizziness and anxiety level indicated that those individuals who reported a reduction in perceived vertigo symptoms also reported a low level of psychiatric symptoms. These findings confirmed the comorbidity of anxiety and dizziness.
